# Epitranscriptomic Control of Drought Tolerance in Rice: The Role of RNA Methylation

**DOI:** 10.3390/plants14132002

**Published:** 2025-06-30

**Authors:** Xiaoru Fan, Yong Zhang, Pengyuan Gu, Misbah Naz

**Affiliations:** 1 School of Chemistry and Life Science, Anshan Normal University, Anshan 114007, China; fanxiaoru@asnc.edu.cn (X.F.); gupengyuan@asnc.edu.cn (P.G.); 2 Liaoning Key Laboratory of Development and Utilization for Natural Products Active Molecules, Anshan 114007, China; 3 Wuxi Branch of Jiangsu Academy of Agricultural, Wuxi 214000, China; 20210074@jaas.ac.cn; 4 State Key Laboratory of Green Pesticide and Guizhou University, Guiyang 550025, China

**Keywords:** *Oryza sativa*, m^6^A RNA methylation, RNA modification, drought stress, drought-signaling pathways, reactive oxygen species, *CRISPR*/*Cas9* technology

## Abstract

Drought stress is a predominant abiotic constraint adversely affecting global rice (*Oryza sativa*) production and threatening food security. While the transcriptional and post-transcriptional regulation of drought-responsive pathways has been widely investigated, the emerging field of epitranscriptomics, particularly RNA chemical modifications such as N6-methyladenosine (m^6^A), adds a new dimension to gene regulation under stress. The most prevalent internal modification in eukaryotic messenger RNA influences RNA metabolism by interacting dynamically with enzymes that add, remove, or recognize the modification. Recent studies in rice reveal that m^6^A deposition is not static but dynamically regulated in response to water-deficit conditions, influencing transcript stability, splicing, nuclear export, and translation efficiency of key drought-responsive genes. This review critically synthesizes current findings on the distribution and functional implications of m^6^A and other epitranscriptomic marks (e.g., 5-methylcytosine [m^5^C], *pseudouridine* [Ψ]) in modulating rice responses to drought. We discuss the regulatory circuitry involving m^6^A effectors such as *OsMTA*, *OsFIP37*, and *YTH* domain proteins and their integration with known drought-signaling pathways including ABA and reactive oxygen species (ROS) cascades. We also highlight emerging high-resolution technologies such as m^6^A-seq, direct RNA sequencing, and nanopore-based detection that facilitate epitranscriptomic profiling in rice. Finally, we propose future directions for translating epitranscriptomic knowledge into crop improvement, including *CRISPR*/*Cas*-based modulation of RNA modification machinery to enhance drought tolerance.

## 1. Introduction

Rice (*Oryza sativa* L.) is one of the most widely cultivated and consumed cereal crops worldwide, serving as a primary food source for over 50% of the global population [1]. However, rice is particularly vulnerable to drought stress due to its semi-aquatic origin and high water requirements during critical growth stages such as flowering and grain filling [2]. Prolonged drought leads to stunted growth, reduced biomass, spikelet sterility, and significant yield loss, posing a serious threat to global food security. Several rice cultivars, such as IR64, Swarna, and Pusa Basmati-1, are known to be highly sensitive to water-deficit conditions [3], while drought-tolerant varieties like N22, Sahbhagi Dhan, and Apo exhibit more robust physiological and molecular responses to stress.

Drought is one of the most damaging abiotic stresses impacting global crop productivity, significantly reducing both yield and quality. Rice, a staple for more than half of the world’s population, is particularly sensitive to drought due to its high water requirements during key developmental stages [4]. As climate change intensifies evapotranspiration and diminishes freshwater availability, drought-induced yield losses in rice have already surpassed those from other environmental stressors combined. Consequently, enhancing drought tolerance in rice is a priority for sustainable agriculture and food security [5]. While extensive research has uncovered key transcriptional, hormonal, and signaling mechanisms underlying drought responses, post-transcriptional regulation via epitranscriptomic modifications has only recently gained attention. Among these, N^6^-methyladenosine (m^6^A) methylation has emerged as a critical RNA modification involved in fine-tuning gene expression during plant stress responses. Historically, efforts to enhance drought tolerance in rice have primarily focused on genetic and transcriptional regulation involving transcription factors, drought-inducible promoters, and hormonal pathways, particularly abscisic acid (ABA) signaling. However, accumulating evidence now highlights the critical role of epitranscriptomic regulation, especially RNA methylation, as a key modulator of gene expression in response to environmental stresses [6].

Among the various RNA modifications, m^6^A is the most prevalent and dynamically regulated internal modification of eukaryotic mRNA. The addition of m^6^A is catalyzed by a conserved multiprotein methyltransferase complex, commonly referred to as the “writer” complex, which includes homologs of Methyltransferase-Like 3 (METTL3), e.g., (*OsMTA*), METTL14, and *Wilms’ Tumor 1-Associating Protein* (WTAP) in rice (*OsFIP37*); enzymes such as ALKBH10B remove methyl groups to enable reversibility, while mainly YTH-domain-containing proteins recognize methylated transcripts and regulate their splicing, stability, translation, and localization within the cell [7].

In rice, recent transcriptome-wide m^6^A profiling under drought stress conditions has revealed that m^6^A deposition is not random but dynamically modulated in response to stress stimuli [8,9]. For instance, drought stress induces selective methylation of transcripts encoding heat shock proteins and transcription factors (e.g., *Dehydration-Responsive Element-Binding protein 2A* (DREB2A); NAC-family proteins, a group of plant-specific transcription factors including *No Apical Meristem* (NAM), (*Arabidopsis Transcription Activation Factor 1*/*2* ATAF1/2), and *Cup-shaped Cotyledon 2* (CUC2); and reactive oxygen species (ROS) scavenging enzymes), thereby enhancing mRNA stability and translation efficiency [10]. Moreover, m^6^A methylation facilitates rapid post-transcriptional responses without requiring new transcription, allowing rice plants to fine-tune gene expression in a timely and energy-efficient manner under stress [11].

Importantly, the pattern and degree of m^6^A methylation vary significantly between drought-sensitive and drought-tolerant rice varieties. Studies have shown that drought-tolerant cultivars maintain more stable m^6^A methylomes and better preserve the expression of protective genes under stress, whereas drought-sensitive cultivars often exhibit m^6^A dysregulation, leading to impaired stress responses [12,13].

This review aims to systematically examine the role of RNA methylation, particularly m^6^A, in shaping drought stress responses in rice [14]. We describe the key components of the m^6^A machinery in rice, highlight the dynamic changes in RNA methylation patterns during drought, and discuss how this epitranscriptomic layer of regulation can be leveraged to breed drought-resilient rice through molecular breeding and genome editing strategies.

## 2. Epitranscriptomic RNA Methylation: A Critical Layer in Drought Stress Adaptation of Rice

Drought stress adversely affects key physiological functions in rice, including reducing stomatal conductance, limiting carbon dioxide (CO_2_) uptake, and impairing photosynthetic efficiency. Drought stress adversely affects key physiological functions in rice by triggering a cascade of water-conservation responses that ultimately hinder photosynthetic performance. One of the earliest responses is a reduction in stomatal conductance, as the stomata close to minimize water loss through transpiration [15]. However, this also limits the diffusion of CO_2_ into the leaf, thereby restricting CO_2_ assimilation in the chloroplasts. As a result, the Calvin cycle is compromised, leading to reduced carbohydrate synthesis and lower biomass accumulation. Moreover, drought-induced oxidative stress and damage to the photosynthetic apparatus, particularly photosystem II, further decrease photosynthetic efficiency. These combined effects not only impair energy production but also disrupt metabolic homeostasis, severely constraining plant growth and productivity under drought conditions [16].

Drought stress impairs chloroplast photochemistry and the efficient utilization of absorbed light energy, leading to a significant reduction in photosynthetic performance and ultimately hampering CO_2_ fixation. If drought or other stress impairs photochemistry, it means the plant’s ability to absorb light, excite electrons, or convert light energy into chemical energy is disrupted, leading to reduced carbon fixation and growth [17]. When drought or other environmental stresses disrupt photochemistry, the plant’s ability to capture light, excite electrons, and generate energy-rich molecules like *Adenosine Triphosphate* (ATP) and *Nicotinamide Adenine Dinucleotide Phosphate* (NADPH) is significantly compromised. This disruption limits the energy supply necessary for the Calvin cycle, where CO_2_ is fixed into carbohydrates. The reduction in carbon fixation leads to lower production of sugars and other essential metabolites, which are crucial for cellular growth, maintenance, and reproduction. Over time, this hampers overall plant biomass accumulation and yield [18]. Furthermore, impaired photochemistry can lead to the overaccumulation of excess light energy, causing the generation of ROS that can damage cellular components unless adequately scavenged by antioxidant systems. Thus, drought-induced impairment of photochemistry not only reduces energy conversion efficiency but also increases oxidative stress, further challenging plant survival and productivity [19]. It impairs chloroplast photochemistry and light energy utilization, ultimately hampering carbon fixation. Being sessile organisms, plants must rely on intricate regulatory networks to perceive and adapt to such stressors while maintaining growth and reproduction [20]. Among these, epigenetic regulation plays a crucial role in mediating gene expression under drought. Within the broader epigenetic framework including DNA methylation, histone modifications, and small RNAs, RNA methylation, particularly m^6^A, has emerged as a critical and reversible epitranscriptomic mark [21]. m^6^A is the most prevalent internal RNA modification in eukaryotes, including *Oryza sativa*, *Arabidopsis thaliana*, and *Nicotiana benthamiana*. It is enriched at RRACH (R = purine; H = A/C/U) and plant-specific UGUA motifs, especially near 3′ untranslated regions (3′ UTRs), stop codons, and transcription start sites [22].

In rice, as in *A. thaliana*, m^6^A methylation influences transcript stability, alternative splicing, nuclear export, and translation efficiency, playing a vital role in drought adaptation (Table 1) [7]. This modification is catalyzed by a conserved “writer” complex, comprising *METTL3* (MTA homolog), METTL14 (MTB homolog), FIP37 (WTAP homolog), *VIRILIZER* (KIAA1429 homolog), and HAKAI components with putative homologs in rice that shape its drought-responsive m^6^A landscape [23]. Demethylation is mediated by “eraser” enzymes such as *ALKBH9B* and *ALKBH10B* (identified in *A. thaliana*), which allow dynamic reversibility. “Reader” proteins like ECT2, ECT3, and ECT4 interpret m^6^A marks, directing methylated transcripts toward degradation or enhanced translation, enabling a rapid and flexible stress response. Transcriptome-wide analyses using *MeRIP-seq* and *m^6^A-seq* in drought-stressed rice have revealed dynamic m^6^A modifications in transcripts encoding transcription factors, ABA signaling components, and other stress-responsive proteins [24].

**Figure 1:**
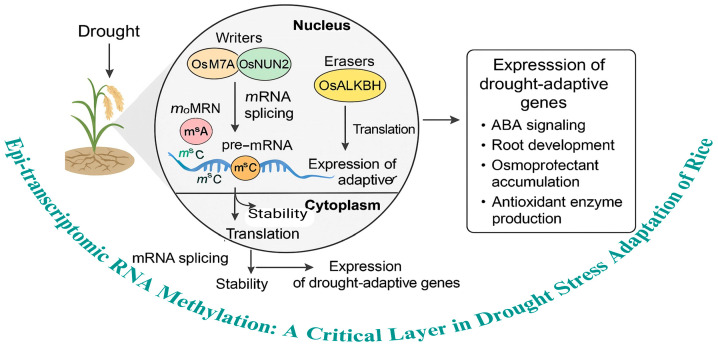
The Role of RNA methylation in epitranscriptomic regulation during rice drought stress adaptation.

Similar findings in *A. thaliana* and tobacco underscore m^6^A’s conserved role in stabilizing or destabilizing mRNAs involved in stress adaptation. Despite its importance, m^6^A detection remains technically challenging. Immunoprecipitation-based methods such as MeRIP-seq and m^6^A-seq offer transcriptome-scale insights but lack single-nucleotide resolution [25]. While the limitations of m^6^A detection methods such as MeRIP-seq and miCLIP are acknowledged, a more comprehensive discussion is needed on how these technical constraints impact the interpretation of methylation data and the identification of biologically functional m^6^A sites [26]. MeRIP-seq offers transcriptome-wide coverage but suffers from low resolution (typically ~100 nucleotides), making it difficult to precisely localize methylation sites and potentially leading to ambiguous associations with regulatory elements such as stop codons or 3′ UTRs. miCLIP, though capable of single-nucleotide resolution, is technically demanding and prone to crosslinking artifacts and biases related to antibody specificity [27]. These limitations can complicate downstream analyses, including motif enrichment studies, correlation with gene expression changes, and integration with other omics datasets. A more detailed explanation from the authors on how such methodological constraints may lead to false positives/negatives or misinterpretation of m^6^A’s functional roles would significantly strengthen the manuscript’s discussion of data reliability and biological relevance [28]. Advanced techniques like methylation-iCLIP (miCLIP) achieve base-resolution mapping by capturing crosslinking-induced mutations during reverse transcription [29]. These technologies have elucidated both conserved and species-specific m^6^A landscapes critical for drought stress regulation in rice (Figure 1) [30].

**Table 1:** Core components of the m^6^A machinery and their putative functions in rice under drought stress.

Component Type	Gene Name (Rice)	*Arabidopsis* Homolog	Putative Function	Known/Proposed Role in Drought Stress	Reference
Writer	OsMTA	AtMTA (METTL3)	Catalytic subunit of the m^6^A methyltransferase complex	May regulate drought-responsive transcripts via m^6^A deposition; expression modulated under abiotic stress	[31,32]
Writer	OsMTB	AtMTB (METTL14)	Forms heterodimer with OsMTA; provides structural support	Facilitates target specificity of OsMTA; role in drought not yet clarified	[33]
Writer	OsFIP37	FIP37	Adaptor protein linking MTA-MTB complex to RNA targets	Essential for embryogenesis in Arabidopsis; drought role in rice unknown	[33]
Eraser	OsALKBH2	ALKBH9B/10B	m^6^A demethylase	Potentially removes m^6^A from transcripts to modulate gene expression under stress; deregulated under stress	[13,34]
Reader	OsECT2	ECT2	YTH-domain-containing protein that binds m^6^A-modified RNAs	May regulate transcript stability and translation during drought	[35]
Reader	OsYTHDF1-like	YTHDF1/2/3	Cytoplasmic reader of m^6^A marks	Controls translation efficiency and mRNA decay of stress-responsive genes	[35,36,37]

This table summarizes current knowledge and gaps regarding the rice m^6^A machinery, useful for highlighting future gene-targeting strategies.

The figure illustrates the role of epitranscriptomic RNA methylation, particularly m^6^A, as a critical regulatory layer in rice adaptation to drought stress. Under drought conditions, specific methyltransferase enzymes such as OsMTA7 and OsNUN2 catalyze the addition of methyl groups (m^6^A and m^5^C) on mRNA transcripts. These modifications occur at various stages, including in pre-mRNA during splicing and in mature mRNA. The methylation enhances mRNA stability and translation efficiency in the cytoplasm, facilitating the expression of drought-adaptive genes. Conversely, *OsALKBH* enzymes remove methyl groups, ensuring the dynamic and reversible nature of this epitranscriptomic mark. This balance between writers and erasers regulates the expression of genes involved in key drought response pathways, including ABA signaling, root development, osmoprotectant accumulation, and antioxidant enzyme production. Collectively, these molecular processes support enhanced drought tolerance in rice by fine-tuning gene expression at the RNA level. This dynamic regulation of mRNA through methylation and demethylation underscores the importance of epitranscriptomic modifications as a critical adaptive mechanism in plants under abiotic stress.

Several core components of the m^6^A methylation machinery have been identified in rice, with homologs in *A. thaliana* showing conserved roles in stress response (Table 1). For instance, *OsMTA*, homologous to *AtMTA* (METTL3), serves as the catalytic subunit of the methyltransferase complex and is known to be modulated under abiotic stress. *OsMTB* (METTL14-like) interacts with *OsMTA* to ensure target specificity, although its direct involvement in drought stress adaptation in rice remains to be clarified. Additionally, proteins such as OsFIP37 and *OsALKBH2* may act as RNA-binding adaptors and demethylases, respectively, suggesting a dynamic regulation of m^6^A deposition and removal in stress-responsive mRNAs. Reader proteins like *OsECT2* and *OsYTHDF1*-like further influence transcript fate post methylation, regulating their stability and translation efficiency (Table 1).

### 2.1. Overview of RNA Methylation in Plants: Types, Distribution, and Detection Technologies

RNA methylation is a crucial epitranscriptomic modification that plays a significant role in regulating RNA stability, processing, and overall function in plants [38,39]. Among the various types of RNA modifications identified, N^6^-methyladenosine (m^6^A) is the most prevalent internal modification found within messenger RNA (mRNA). Other important modifications include 5-methylcytosine (m^5^C) and pseudouridine (Ψ), which occur in a wide range of RNA species such as transfer RNA (tRNA), ribosomal RNA (rRNA), and long non-coding RNAs [40]. These modifications collectively influence numerous biological processes by modulating RNA metabolism and gene expression. In plants, m^6^A methylation occurs predominantly at conserved sequence motifs such as RRACH (where R is a purine and H is A, C, or U) and the plant-specific UGUA motif [22]. This modification is enriched near critical transcript regions including stop codons, 3′ untranslated regions (3′UTRs), and transcription start sites, where it regulates mRNA splicing, stability, export, and translation. Meanwhile, m^5^C is primarily detected in tRNAs and rRNAs but has recently been observed in mRNAs, where it affects RNA stability and translation efficiency. *Pseudouridine*, formed by isomerization of uridine, contributes to RNA structural stability and enhances translation fidelity [4].

The distribution and abundance of these methylations vary across plant species and tissues, reflecting their specific roles in development and environmental responses. For instance, studies in model plants like *A. thaliana* and key crops such as rice (*O. sativa*) and tobacco (*N. benthamiana*) reveal conserved yet species-specific methylation patterns that underpin their adaptive capabilities [22]. Recent technological advances have greatly enhanced our ability to detect and map RNA methylation, deepening our understanding of epitranscriptomic regulation in plants. Commonly used methods like methylated RNA immunoprecipitation sequencing (MeRIP-seq) and m^6^A-seq allow researchers to profile methylation across the transcriptome, though they lack precise single-nucleotide resolution [41]. To address this limitation, techniques such as methylation individual-nucleotide-resolution crosslinking and immunoprecipitation (miCLIP) have been developed, enabling base-resolution mapping of m^6^A sites by detecting antibody-induced mutations during reverse transcription. Emerging tools like nanopore direct RNA sequencing, which does not require reverse transcription, and chemical labeling approaches for m^5^C and *pseudouridine* are further expanding the scope and accuracy of RNA modification detection. Collectively, these technologies are revealing the dynamic and complex roles of RNA methylations in plant stress responses, including critical adaptations to drought conditions [42].

#### 2.1.1. Translational Potential of Epitranscriptomics in Crop Improvement

Recent advances in understanding the role of m^6^A RNA methylation in plant stress responses have opened new avenues for crop improvement [43]. Functionally validated m^6^A-associated genes, particularly those involved in the regulation of drought-responsive transcription factors (e.g., *DREB*, *NAC*, *bZIP* families), ABA signaling components, and antioxidant enzymes, represent promising candidates for molecular breeding [44]. These genes and their epitranscriptomic modifications can serve as molecular markers for marker-assisted selection (MAS), enabling breeders to screen and select varieties with enhanced drought tolerance more efficiently [45].

Furthermore, the rapid progress in genome editing technologies, such as *CRISPR*/*Cas* systems, offers opportunities to precisely manipulate m^6^A-related genes or the m^6^A consensus motifs on target transcripts [29]. Editing m^6^A writer (e.g., *METTL3* homologs), eraser (e.g., *ALKBH* homologs), or reader proteins can modulate the m^6^A landscape, thereby stabilizing or enhancing the translation of key stress-responsive mRNAs. Such targeted epitranscriptomic engineering can improve plant resilience without altering the underlying DNA sequence significantly, offering a novel layer of genetic regulation to exploit [46].

Integration of epitranscriptomic data into genomic selection (GS) models could further enhance breeding accuracy by capturing regulatory variation not detectable at the DNA sequence level alone. Multi-omics datasets incorporating m^6^A profiles alongside genomics, transcriptomics, and phenomics can provide a comprehensive framework for predicting drought tolerance phenotypes [47,48]. Proof-of-concept studies in model plants have demonstrated that overexpression of m^6^A writers or readers can enhance stress tolerance by improving mRNA stability and translation of drought-related genes. Translating these findings to crops holds considerable promise but requires careful functional validation and field evaluation [49]. Overall, leveraging the translational potential of epitranscriptomics offers a powerful complement to traditional and molecular breeding approaches, accelerating the development of drought-resilient crop varieties to meet the challenges of climate change and food security [50].

#### 2.1.2. N^6^-Methyladenosine (m^6^A): A Key Epitranscriptomic Mark

**Figure 2:**
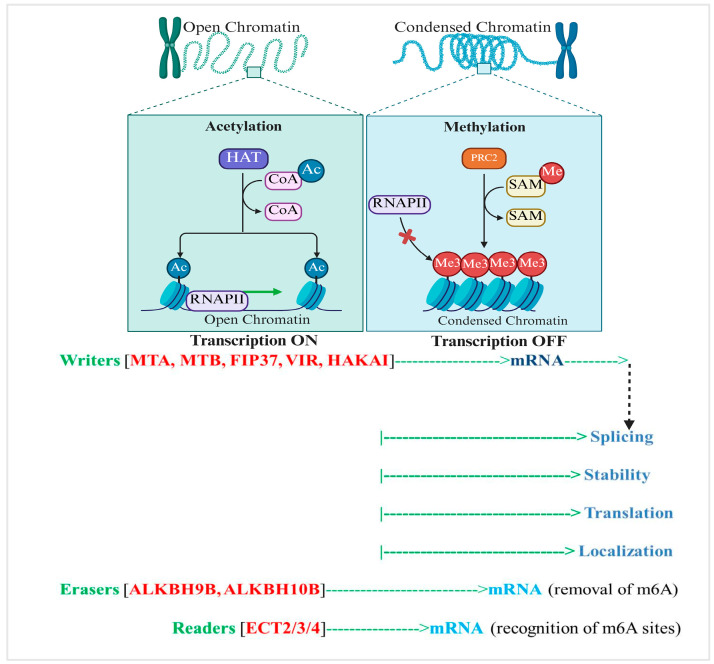
Overview of m^6^A RNA methylation machinery in plants.

N^6^-methyladenosine (m^6^A) is the most prevalent internal modification found in eukaryotic messenger RNA (*mRNA*), playing a crucial role in regulating RNA metabolism and gene expression (Figure 2). This dynamic and reversible epitranscriptomic mark affects multiple aspects of RNA function, including stability, translation, splicing, and localization, thus influencing vital biological processes such as plant responses to environmental stresses like drought. m^6^A modifications predominantly occur within conserved sequence motifs, most notably the RRACH consensus (where R represents purines A or G, and H is A, C, or U). In plants, a distinctive UGUA motif also serves as a methylation site, underscoring species-specific methylation patterns. These methylations are enriched near transcript features such as stop codons, 3′UTRs, and transcription start sites (TSSs), which is fundamental to their role in controlling mRNA, including its stability and translational efficiency. The addition of m^6^A marks is catalyzed by a multicomponent methyltransferase complex, and the key components of this complex include MTA (the METTL3 homolog responsible for catalytic activity), MTB (METTL14 homolog that enhances substrate specificity and complex stability), FIP37 (WTAP homolog guiding the complex to RNA targets), VIRILIZER (KIAA1429 homolog involved in complex assembly), and *HAKAI*, an E3-ubiquitin-ligase-like protein that supports complex integrity. These writer proteins are evolutionarily conserved across eukaryotes, including important plant models such as *A. thaliana* and major crops like *Oryza sativa*, highlighting their indispensable role in epitranscriptomic regulation. Importantly, m^6^A marks are reversible through the action of enzymes, such as ALKBH9B and ALKBH10B in plants, which belong to the AlkB family of dioxygenases that catalyze oxidative demethylation. This reversibility allows plants to dynamically modulate gene expression in response to developmental signals and environmental stresses, including drought. The biological outcomes of m^6^A modifications are mediated by proteins that specifically recognize methylated RNA and influence its fate. In plants, prominent readers include the YTH domain-containing proteins ECT2, ECT3, and ECT4, which bind m^6^A-modified transcripts to regulate mRNA stability, translation, and subcellular localization.

Figure 2 illustrates the core components involved in m^6^A RNA methylation in plants, including the methyltransferase complexes that catalyze the addition of m^6^A marks on RNA molecules. Demethylases remove these modifications, enabling dynamic regulation. Specific proteins recognize and bind m^6^A sites to mediate downstream effects on RNA stability, splicing, and translation. This machinery collectively modulates gene expression and plays critical roles in plant development and stress responses.

These readers enable plants to rapidly adjust gene expression post-transcriptionally under stress conditions by directing the degradation or enhanced translation of specific transcripts. To study m^6^A distribution and function, several high-throughput mapping technologies have been developed. Techniques such as MeRIP-seq and m^6^A-seq use antibody-based enrichment coupled with sequencing to identify m^6^A-enriched regions transcriptome-wide, but lack single-nucleotide precision. The miCLIP method addresses this limitation by employing UV crosslinking and antibody-induced mutations during reverse transcription to achieve single-nucleotide resolution mapping of m^6^A sites. Emerging tools like nanopore direct RNA sequencing enable detection of RNA modifications without the need for reverse transcription or amplification, while chemical labeling strategies enhance the mapping of other RNA methylation marks, collectively advancing our understanding of m^6^A’s dynamic role in plant stress responses, including drought tolerance.

### 2.2. Tools for Mapping m^6^A: MeRIP-Seq, m^6^A-Seq, miCLIP

Mapping m^6^A modifications across the transcriptome relies on specialized high-throughput sequencing techniques that combine antibody-based enrichment with next-generation sequencing. MeRIP-seq (methylated RNA immunoprecipitation sequencing) and m^6^A-seq are widely used methods that immunoprecipitate fragmented RNA using m^6^A-specific antibodies, followed by sequencing to identify methylated regions transcriptome-wide. While these approaches efficiently reveal m^6^A-enriched peaks, their resolution is limited to about 100–200 nucleotides, making it difficult to pinpoint the exact modified adenosine within the enriched region.

To overcome this, miCLIP (methylation individual-nucleotide-resolution crosslinking and immunoprecipitation) was developed. miCLIP utilizes UV-induced crosslinking between the antibody and RNA, causing specific mutations or truncations during reverse transcription that serve as precise markers form m^6^A sites. This enables single-nucleotide-resolution mapping, providing a more detailed and accurate landscape of m^6^A modifications. Emerging technologies, such as nanopore direct RNA sequencing, also show promise for detecting m^6^A modifications directly on native RNA molecules without the need for amplification or conversion, further enhancing epitranscriptomic analysis (Figure 2).

#### Functional Validation of m^6^A RNA Methylation

Functional validation of m^6^A RNA methylation is crucial for understanding its regulatory role in plant drought stress responses. While transcriptome-wide studies such as MeRIP-seq have identified numerous m^6^A-modified genes under drought, these findings remain largely correlative without experimental validation. In *A. thaliana*, genetic studies using loss- and gain-of-function mutants have demonstrated that core m^6^A writers like MTA, MTB, and FIP37 are essential for normal growth and stress adaptation [36,51]. For example, MTA-deficient plants show increased sensitivity to drought, indicating that m^6^A plays a positive role in stress tolerance. Additionally, functional analyses of m^6^A reader proteins, such as ECT2, have revealed their ability to bind methylated transcripts and regulate their stability and translation, further supporting the functional importance of m^6^A in stress-responsive gene expression [52]. More precise techniques, including miCLIP and *CRISPR*-mediated mutagenesis of specific m^6^A sites, have shown that m^6^A modifications can directly influence the fate of target transcripts like *DREB2A* and *RD29A*, which are critical for drought response. Despite these advances, functional validation in rice remains limited. Most rice m^6^A machinery components have been identified based on homology to Arabidopsis genes, but their physiological roles under drought stress have not been experimentally confirmed [53]. Future studies should focus on generating knockout or overexpression lines in rice, performing RNA stability assays, and integrating epitranscriptomic data with other regulatory layers such as histone modifications and DNA methylation to gain a comprehensive understanding of m^6^A-mediated drought adaptation [54].

m^6^A modification has been implicated in regulating various drought adaptation pathways, including transcription factor activity, ABA signaling, and oxidative stress responses (Table 2). ABA improves the efficiency of stress responses by coordinating physiological, molecular, and biochemical pathways enabling plants to survive and recover from adverse environmental conditions with minimal energy cost and maximum resilience [55]. For instance, in *A. thaliana*, m^6^A increases the stability of DREB2A and NAC transcripts, crucial for drought-induced transcriptional reprogramming. However, similar functional validations are lacking in rice. Moreover, components of the ABA signaling cascade such as PYR/PYL receptors and SnRK2 kinases are known m^6^A targets in maize and *A. thaliana*, but their m^6^A-mediated regulation in rice remains poorly characterized. Importantly, the interplay between m^6^A and chromatin-level epigenetic mechanisms, as well as the circadian clock and antioxidant defenses, underscores the epitranscriptomic control over multiple layers of stress adaptation (Table 2).

### 2.3. Role of m^6^A RNA Methylation in Drought Stress Adaptation

m^6^A RNA methylation has emerged as a crucial epitranscriptomic mechanism that modulates plant responses to drought stress by fine-tuning gene expression at multiple regulatory levels. One key role of m^6^A is its influence on the expression of transcription factors (TFs) that orchestrate drought-responsive pathways [63]. By affecting the stability and translation efficiency of mRNAs encoding TFs such as DREB, NAC, and MYB families, m^6^A ensures rapid and dynamic control of the transcriptional programs necessary for stress adaptation. This regulation allows plants to activate defense genes promptly without permanently altering their genome [64]. In addition to transcription factors, m^6^A methylation modulates components of the ABA signaling pathway, which is central to drought response. ABA mediates stomatal closure, osmotic adjustment, and expression of protective proteins under drought. Studies have shown that m^6^A marks regulate the transcript levels and translational output of ABA receptors, signaling intermediates, and downstream effectors, thereby fine-tuning ABA sensitivity and response intensity [65]. This epitranscriptomic control helps plants balance growth and stress tolerance efficiently. Beyond individual genes, m^6^A exerts broad regulation over complex stress-responsive gene networks. By selectively methylating transcripts involved in reactive oxygen species (ROS) detoxification, osmolyte biosynthesis, and cellular repair, m^6^A shapes the overall gene expression landscape that underpins drought resilience. This systemic regulation ensures coordinated activation of protective pathways while preventing unnecessary energy expenditure [66].

Mechanistically, m^6^A impacts transcript stability, alternative splicing, and translation during drought stress. Methylated RNAs may exhibit altered degradation rates, resulting in either stabilization or rapid turnover depending on the context and the associated reader proteins [67]. m^6^A can influence splicing patterns to generate stress-specific isoforms, and enhance or repress translation of key drought-responsive proteins. These multifaceted roles enable plants to dynamically reprogram their proteome in response to fluctuating water availability. Comparative studies in *A. thaliana* and other model plants have provided valuable insights into the conserved and species-specific aspects of m^6^A-mediated drought adaptation [68]. While the core machinery and many target transcripts are conserved, variations in methylation patterns and reader protein functions contribute to differential drought tolerance phenotypes. This comparative knowledge is critical for translating fundamental epitranscriptomic discoveries into crop improvement strategies for drought resilience [69].

## 3. Dynamic m^6^A Methylation Landscape Under Drought in Rice

Recent transcriptome-wide studies have begun to unravel the dynamic changes in m^6^A RNA methylation profiles in rice subjected to drought stress [46]. Using techniques like MeRIP-seq and miCLIP, researchers have mapped thousands of m^6^A sites across the rice transcriptome, revealing a global remodeling of methylation patterns under water-deficit conditions. These studies highlight that drought stress triggers both gain and loss of m^6^A marks on specific sets of mRNAs, suggesting that m^6^A modification acts as a rapid and reversible layer of gene regulation that complements transcriptional responses [46].

Temporal and spatial profiling of m^6^A under drought further reveals that methylation patterns vary significantly with the duration of stress and among different tissues [70]. For instance, early drought exposure may induce m^6^A modifications in transcripts involved in signaling and transcriptional regulation, while prolonged stress targets genes related to cellular protection and metabolic adjustments [71]. Tissue-specific differences also exist; roots often show distinct m^6^A remodeling compared to leaves, reflecting their unique roles in water sensing and conservation. This spatiotemporal plasticity underscores m^6^A’s role as a finely tuned regulator adapting to complex drought scenarios [72].

Functional enrichment analyses of drought-responsive m^6^A-modified transcripts consistently point to categories involved in stress signaling, hormone pathways (notably ABC), osmoprotection, and ROS scavenging. Genes associated with these processes are often methylated to modulate their expression post-transcriptionally, thereby enhancing the plant’s ability to mitigate drought-induced damage. This selective m^6^A methylation ensures prioritized regulation of critical pathways required for survival under limited water availability [73].

Importantly, changes in the m^6^A methylation landscape correlate strongly with physiological and metabolic responses observed in drought-stressed rice [46]. Enhanced m^6^A modification of transcripts involved in stomatal closure, root architecture remodeling, and osmolyte accumulation coincide with observed phenotypic traits such as reduced transpiration, deeper rooting, and improved cellular osmotic balance [74]. This correlation demonstrates that m^6^A-mediated post-transcriptional regulation is integral to linking environmental cues to adaptive physiological mechanisms, ultimately influencing drought tolerance at the whole-plant level [34].

### 3.1. Cross-Talk Between RNA Methylation and Other Regulatory Pathways

#### 3.1.1. Interplay with Small RNAs (miRNAs, siRNAs)

**Figure 3:**
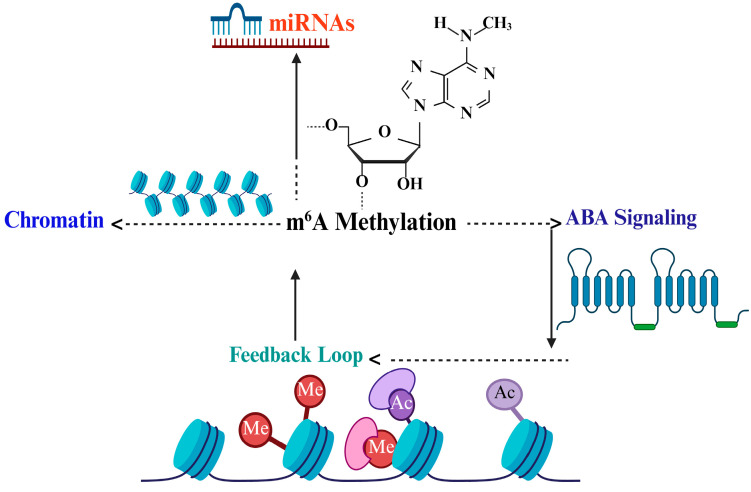
Cross-talk between m^6^A methylation and other regulatory pathways.

RNA methylation, particularly m^6^A, interacts closely with small RNA pathways to orchestrate gene expression under drought stress (Figure 3) [4]. m^6^A modifications can influence the biogenesis and stability of microRNAs (miRNAs) and small interfering RNAs (siRNAs), which are crucial post-transcriptional regulators [75]. For example, m^6^A methylation of primary miRNA transcripts may affect their processing efficiency by Dicer-like enzymes, altering the pool of mature miRNAs that target stress-responsive genes [4]. Conversely, small RNAs can regulate the expression of RNA methylation machinery components, creating a regulatory feedback loop. This cross-talk ensures coordinated fine-tuning of gene silencing and activation during drought adaptation [76].

Figure 3 depicts the key molecular components responsible for adding, removing, and interpreting m^6^A modifications on RNA in plants. The methyltransferase complex catalyzes the methylation of adenosine residues, marking RNA molecules with m^6^A. Demethylase enzymes can reverse this modification, allowing dynamic regulation of RNA function. Specialized reader proteins recognize these m^6^A marks and influence RNA processes such as splicing, stability, export, and translation. Together, these components form a regulatory network that controls gene expression and impacts various physiological processes including growth, development, and responses to environmental stress in plants like *A. thaliana* [77].

#### 3.1.2. Interaction with Chromatin Modifiers and Histone Marks

RNA methylation pathways do not operate in isolation but interact with chromatin-level regulators to achieve integrated control of gene expression [78]. Evidence suggests that m^6^A-modified transcripts can influence chromatin state indirectly by modulating the expression or activity of chromatin remodelers and histone-modifying enzymes [79]. This interplay facilitates coordinated regulation at both transcriptional and post-transcriptional levels, enhancing the plasticity of gene expression programs under drought stress [80].

#### 3.1.3. Coordination with Transcriptional and Translational Controls

m^6^A RNA methylation acts as a bridge between transcriptional regulation and translational control, modulating the flow of genetic information in response to drought [4]. While transcription factors control the initial mRNA synthesis, m^6^A modifications influence mRNA splicing, nuclear export, stability, and translation efficiency [7]. This coordination allows plants to rapidly adjust protein synthesis independently of transcriptional changes, which is crucial during acute stress. By affecting mRNA fate, m^6^A ensures that transcripts encoding key drought-responsive proteins are timely translated or degraded, optimizing resource allocation and cellular homeostasis [81].

#### 3.1.4. Potential Feedback Loops Between ABA Signaling and RNA Methylation

ABA is a central hormone in drought response, and emerging evidence highlights bidirectional feedback between ABA signaling and RNA methylation pathways [81]. ABA can modulate the expression and activity of m^6^A writers, erasers, and readers, altering the epitranscriptomic landscape to favor drought tolerance. In turn, m^6^A modifications regulate transcripts involved in ABA biosynthesis, signaling components such as receptors and transcription factors, thus fine-tuning hormone sensitivity and response amplitude [75]. These feedback loops create a dynamic regulatory network allowing plants to adaptively recalibrate ABA signaling according to stress severity and duration, ensuring robust yet flexible drought tolerance mechanisms [67].

## 4. Critical Issues and Research Gaps

Despite rapid advances in understanding RNA modifications, particularly m^6^A, several critical issues and research gaps remain in elucidating their role in drought tolerance in rice [61]. One major limitation is the insufficient functional characterization of the core m^6^A machinery including writers, erasers, and readers in rice. Although these components have been identified based on homology with *A. thaliana*, their specific roles under drought stress have not been thoroughly explored. There is a notable absence of mutant or overexpression lines in rice that could help define their contributions to drought adaptation [82].

Furthermore, a comprehensive understanding of their spatial expression patterns and regulatory dynamics in response to drought is lacking. Another key gap lies in the incomplete identification of drought-responsive m^6^A-modified transcripts in rice. While some studies have applied MeRIP-seq or m^6^A-seq in general stress contexts, few have examined the transcriptome-wide m^6^A landscape specifically under drought, particularly with high resolution or temporal depth [83]. This limits our understanding of how m^6^A marks shift over the course of drought stress, and there is minimal insight into tissue- or cell-type-specific methylation changes. Additionally, integration of m^6^A methylation data with transcriptomics, proteomics, and physiological traits remains underdeveloped, restricting the identification of functionally relevant targets [84].

The biological consequences of m^6^A modifications in rice drought response also remain ambiguous. Whether m^6^A predominantly stabilizes or destabilizes transcripts, or how it modulates splicing and translation of specific mRNAs under drought stress, is not well established [4]. These mechanisms are often inferred from model systems rather than directly demonstrated in rice, leaving a knowledge gap regarding their true regulatory outcomes. This gap is compounded by the limited investigation into how m^6^A interacts with other regulatory layers such as small RNAs, histone modifications, or hormone signaling pathways [85]. In particular, the cross-talk between m^6^A methylation and ABA biosynthesis and signaling central to drought response is poorly understood. There is also limited information on how m^6^A might influence miRNA activity or interact with the chromatin landscape in a stress-responsive manner [86].

From an applied perspective, epitranscriptomic insights have yet to be translated into breeding strategies or practical applications for drought-resilient rice. There are currently no established biomarkers based on RNA methylation for selecting drought-tolerant cultivars [87]. Tools for precise epigenome editing of m^6^A marks in rice are still in early stages, and natural genetic variation in m^6^A machinery across rice varieties remains largely unexplored. Finally, technical constraints continue to hinder progress, as commonly used m^6^A mapping methods like MeRIP-seq lack single-nucleotide resolution, which is crucial for identifying functional methylation sites [88]. Low abundance of modified transcripts under specific conditions may also obscure key targets. To overcome these limitations, multi-omics platforms integrating RNA-seq, m^6^A profiling, ribosome footprinting, and metabolic data are urgently needed to create a holistic view of m^6^A-mediated drought response in rice [89].

## 5. Future Research Directions

▪ To fully realize the potential of RNA methylation as a target for improving drought tolerance in rice, several key research directions must be prioritized. First, functional genomics studies are urgently needed to elucidate the roles of RNA methylation regulatory proteins writers, erasers, and readers using *CRISPR*/*Cas9*-based gene editing, overexpression, or knockdown approaches. Particular attention should be given to tissue-specific and stress-inducible expression patterns to uncover spatial and temporal regulation under drought conditions.
▪ Second, high-resolution mapping of RNA modifications using techniques such as miCLIP or nanopore-based direct RNA sequencing should be extended to drought-treated rice plants. These studies should include time-course analyses and organ-specific profiling to capture how the modification landscape dynamically changes during drought progression and recovery. Integrating these data with transcriptomic, proteomic, and metabolomic datasets will be essential for identifying functionally significant target genes and pathways.
▪ Third, mechanistic studies are needed to dissect the functional effects of RNA methylation on RNA stability, alternative splicing, nuclear export, and translational efficiency under drought stress. Additionally, the cross-regulatory interactions between RNA methylation, small RNAs, histone modifications, and phytohormone signaling, particularly ABA, require detailed investigation using multi-omic and genetic approaches.
▪ Fourth, future efforts should focus on translating epitranscriptomic insights into breeding programs by identifying natural allelic variations in RNA methylation regulators across diverse rice germplasms. The development of epigenome-editing tools to manipulate RNA modifications in a site-specific manner could open new avenues for precision crop improvement. Moreover, the identification of RNA modification-based biomarkers associated with drought resilience may facilitate marker-assisted selection in breeding pipelines.
▪ Fifth, multi-omics approaches including epitranscriptomics (e.g., m^6^A-seq), transcriptomics (RNA-seq), proteomics, metabolomics, and chromatin accessibility assays (ATAC-seq or ChIP-seq) can be integrated to achieve a systems-level understanding of plant drought responses. By correlating m^6^A modifications with changes in transcript abundance, translation efficiency (assessed via ribosome profiling), and protein or metabolite levels, researchers can identify key regulatory nodes that coordinate stress adaptation. However, the integration of these diverse data types presents significant methodological and analytical challenges, such as ensuring high spatial and temporal resolution, effectively normalizing heterogeneous datasets, and applying advanced machine-learning-based network inference models to unravel complex regulatory interactions.

## 6. Conclusions

RNA methylation, especially m^6^A, adds a crucial regulatory layer to plant stress responses, with emerging evidence pointing to its role in modulating drought tolerance in rice. While the current understanding remains preliminary and largely correlative, advancing technologies such as high-resolution methylome mapping and functional validation of m^6^A regulators will be key to unlocking its full potential. As research progresses, epitranscriptomic regulation may offer innovative strategies for breeding drought-resilient rice varieties, supporting sustainable agriculture in the face of climate change.

## Figures and Tables

**Table 2:** Roles of m^6^A RNA methylation in drought adaptation pathways in plants.

Regulatory Level	Example Targets or Pathways	Impact of m^6^A Modification	Evidence (Species)	Identified Gap in Rice	Reference
Transcription factor regulation	*DREB2A*, *NAC*, *bZIP TFs*	m^6^A increases mRNA stability or enhances translation	*Arabidopsis thaliana*, tomato	Functional validation of TFs under m^6^A control is lacking	[56]
ABA signaling pathway	*PYR*/*PYL*/*RCAR*, *PP2Cs*, *SnRK2s*	Fine-tunes ABA response by modulating key mRNA levels	*A. thaliana*, maize	ABA components under m^6^A control in rice not fully mapped	[57]
Gene networks	LEA proteins, HSPs, RD29A	Stabilizes stress-inducible transcripts	*A. thaliana*	Transcriptome-wide validation in rice not yet available	[58]
Epigenetic–epitranscriptomic interface	Histone-modifying enzymes, chromatin remodelers	Co-regulation of gene expression with histone marks and RNA methylation	*A. thaliana*	Epigenomic integration with m^6^A data is absent in rice	[54]
Oxidative stress response	SOD, CAT, APX (antioxidant enzymes)	Modulates ROS-scavenging enzyme transcripts	Wheat, *A. thaliana*	Role of m^6^A in rice oxidative stress management remains unexplored	[59]
Protein translation efficiency	eIFs (eukaryotic initiation factors), ribosomal proteins	Regulates translation under stress	Human cells, *A. thaliana*	No direct evidence in rice under drought	[60]
Circadian clock regulation	CCA1, TOC1 (circadian clock genes)	Alters mRNA turnover of clock-related genes	*A. thaliana*	Potential link between m^6^A and drought-responsive circadian shifts in rice is unknown	[61]
Long non-coding RNAs (lncRNAs)	Drought-responsive lncRNAs	m^6^A marks influence lncRNA stability and function	Maize, *A. thaliana*	No studies on m^6^A-modified lncRNAs in rice drought response	[62]
Alternative polyadenylation	Poly(A) site selection regulators	Affects mRNA stability and processing efficiency	Mammalian cells, *A. thaliana*	Whether m^6^A influences polyadenylation in rice under drought is unclear	[63]

This table summarizes the core components of the m^6^A RNA methylation machinery identified in rice (*Oryza sativa*), including homologs characterized in *A. thaliana*, along with their putative functions and potential involvement in drought stress responses. methyltransferase enzymes that install the m^6^A mark, demethylases that remove it, and RNA-binding proteins that interpret it affect RNA fate. While these components have been bioinformatically or functionally inferred from *A. thaliana* and mammalian studies, their precise regulatory mechanisms under drought stress in rice remain largely uncharacterized and require further functional validation.

## Data Availability

All data generated or analyzed during this study are included in this published article.

## Abbreviations

m^6^A	N^6^-Methyladenosine
ABA	Abscisic Acid
MeRIP-seq	Methylated RNA Immunoprecipitation Sequencing
miCLIP	Methylation Individual-Nucleotide-Resolution Crosslinking and Immunoprecipitation
YTH	YT521-B Homology Domain
METTL3	Methyltransferase-Like 3
METTL14	Methyltransferase-Like 14
3′UTR	3′ Untranslated Region
FIP37	FKBP12 Interacting Protein 37 (WTAP homolog in plants)
TF	Transcription Factor
